# Primary stability of dental implants placed by novice surgeons: an ex vivo study across two implant systems in a porcine mandible model

**DOI:** 10.1186/s40729-026-00727-w

**Published:** 2026-11-02

**Authors:** Juliane Wagner, Lais Ghafoor, Julia Baas, Dorothee Spille, Paul Kübel, Felix Lorich, Ralf Smeets, Aydin Gülses, Jörg Wiltfang, Johannes Spille

**Affiliations:** 1 Department of Oral and Maxillofacial Surgery, Christian-Albrechts-University, University Hospital of Schleswig-Holstein, UKSH Campus Kiel, Arnold-Heller-Straße 3, 24105 Kiel, Germany; 2 Department of Neurosurgery, University Medical Center Hamburg- Eppendorf, Hamburg, Germany; 3 Department of Oral and Maxillofacial Surgery, University Medical Center Hamburg-Eppendorf, Hamburg, Germany

**Keywords:** Dental implants, Primary stability, Implant stability quotient, Resonance frequency analysis, Surgical experience, Porcine mandible, Implant training

## Abstract

**Purpose:**

Dental implant placement is a technically demanding procedure in which surgical experience is considered a key determinant of outcome. Primary stability, as measured by the implant stability quotient (ISQ) via resonance frequency analysis (RFA), is a critical predictor of successful osseointegration. This study aimed to quantify the influence of surgical experience on primary implant stability in a standardized porcine mandible model.

**Methods:**

Dental students with no prior implant experience and experienced oral surgeons placed implants using two commercially available systems (Straumann Standard Plus and Astra Tech EV). Primary stability was assessed by RFA immediately after insertion at four standardized measurement positions. ISQ values were analyzed using a linear mixed-effects model with surgical experience, implant system, and measurement position as fixed factors and a random intercept for implant to account for the non-independence of the four measurements obtained per implant.

**Results:**

A total of 760 ISQ measurements were included in the analysis. Experienced surgeons achieved significantly higher ISQ values (M = 75.72, SD = 5.22) than students (M = 68.68, SD = 11.61; *p* < 0.001), with an overall mean difference of approximately 7 ISQ points. Within the Straumann Standard Plus, the difference was 8.14 ISQ points (*p* = 0.028); within the Astra Tech EV, the difference was 5.42 ISQ points (*p* < 0.001). Variability was markedly greater in the student group across both systems.

**Conclusions:**

Surgical experience significantly influences primary implant stability within both implant systems investigated. Novice operators achieve lower and less consistent ISQ values, which may have clinical implications for the selection of loading protocols. These findings suggest that structured training may help improve primary stability among less-experienced operators, a hypothesis that warrants prospective clinical evaluation.

**Clinical trial number:**

Not applicable.

## Background

Missing teeth due to caries, periodontitis, or trauma represent a widespread clinical problem. Rehabilitation with dental implants has demonstrated superior outcomes compared to conventional prostheses in terms of chewing comfort, appearance, and patient quality of life, but is associated with higher costs and the risk of implant failure [1, 2].

Successful implant integration depends on both biological and biomechanical factors. Among the latter, primary stability — the immediate mechanical fixation of the implant in bone at the time of placement — is considered a critical prerequisite for osseointegration and directly influences the choice of loading protocol. Primary stability is commonly assessed using the implant stability quotient (ISQ) via resonance frequency analysis (RFA) or insertion torque values (ITV). ISQ evaluates the axial and lateral stiffness of the bone-implant interface and is less dependent on cutting resistance than ITV, making it more reflective of the system’s composite elastic properties. ISQ values exceeding 60 are generally considered favorable, and ITV is typically targeted between 25 and 40 Ncm [3, 4]. The evidence on the correlation between ITV and ISQ is heterogeneous, and neither parameter alone fully determines implant survival; both contribute to the clinical assessment of primary stability and the decision on loading timing [5, 6].

Meeting these parameters can be difficult due to various factors, such as low bone density or bony defects [7]. In lower bone density (D3 to D4), as reported by Misch et al., implants with a more aggressive thread pitch or underdrilling procedures showed better primary stability than standard implant designs and operating procedures [8–11]. Similarly, a limited amount of surrounding bone can cause lower primary stability. In these cases, highly trained surgeons showed lower failure rates than inexperienced surgeons [10].

However, the specific influence of surgical experience on primary stability has rarely been quantified under standardized conditions. This study aims to assess and compare the primary stability achieved by dental students placing their first implants with that of experienced oral surgeons, using two different implant systems in a porcine mandible model. The ISQ, measured by resonance frequency analysis immediately after insertion, served as the primary outcome parameter.

## Materials and methods

### Study design

This experimental ex vivo study was conducted in a standardized laboratory setting, using fresh porcine mandibles as a bone-surrogate model. 135 dental students in their final year of clinical training, with no prior implant placement experience, and two experienced oral and maxillofacial surgeons participated in the study. The two experienced surgeons were characterized as follows: Surgeon 1 had approximately 25 years of implant experience with an estimated > 5000 implants placed, and Surgeon 2 had approximately 7 years of experience with an estimated > 1000 implants placed. Both surgeons routinely place the two implant systems investigated. Each dental student placed a single implant, whereas the two experienced surgeons together placed the remaining implants. In total, 190 implants were placed (Straumann Standard Plus: 70 implants, comprising 56 placed by students and 14 by surgeons; Astra Tech EV: 120 implants, comprising 79 placed by students and 41 by surgeons). Because each student placed only one implant, no within-student learning-curve effect could arise in the student group. Each operator placed implants using a commercially available system: the Straumann Standard Plus 4.1 × 10 mm (Straumann Holding AG, Basel, Switzerland) or the Astra Tech EV 4.2 × 13 mm (Dentsply Sirona Inc., Charlotte, North Carolina, USA). All implants were placed according to the respective manufacturer’s drilling protocols. The porcine mandibles were obtained from a regionally certified butcher, and the cold chain was maintained without interruption from procurement to experimental use. The mandibles were used within two days after slaughter. All specimens were allowed to equilibrate to room temperature before implant placement and measurement. Implants were placed in the same edentulous premolar of each mandible (a new mandible was used for each implant).

**Fig. 1:**
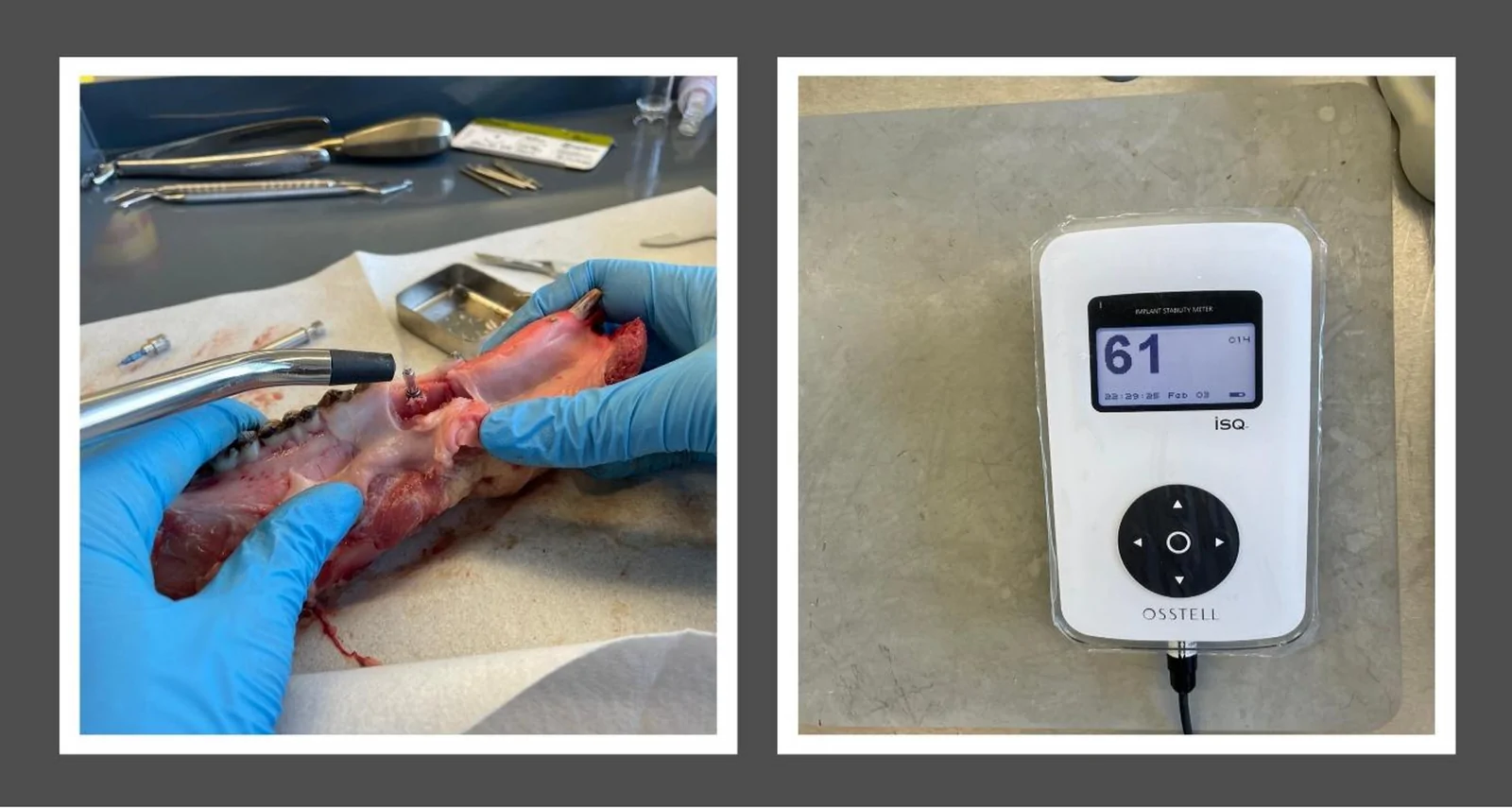
Experimental setup. On the left side, a dental implant has been inserted into the porcine mandible. Measurements are taken from mesial, distal, buccal, and lingual. On the right side, the ISQ value is displayed on the Osstell ISQ device

Primary stability was assessed immediately after implant insertion using resonance frequency analysis (RFA) with the Osstell ISQ device (Osstell AB, Gothenburg, Sweden) using a SmartPeg transducer specific to each implant system (Straumann Standard Plus: Type 4; Astra Tech EV: Type 32) (Fig. 1). ISQ values were recorded at four standardized positions around the implant neck: mesial, distal, buccal (vestibular), and lingual. All measurements were performed by a single trained investigator to minimize inter-rater variability.

Ethical approval was obtained from the Ethics Commission of the Faculty of Medicine at the Christian-Albrechts-University, Kiel (D 604/25), and the study was conducted in accordance with the Declaration of Helsinki.

No a-priori sample-size calculation was performed; the available sample size was determined by the number of dental students enrolled in the practical course and the implants placed by the two experienced surgeons during the same period.

The dental implants and the Osstell ISQ device used in this study were used for student training and are the clinic’s property. The manufacturers had no role in the study design, data collection, analysis, interpretation, or the decision to submit for publication.

### Statistical analysis

Statistical analyses were performed using SPSS 31.0 (IBM Corp., Armonk, NY, USA). The ISQ was defined as the dependent variable. To account for the non-independence of the four ISQ measurements obtained per implant, a linear mixed-effects model was fitted with surgical experience (student vs. surgeon), implant system (Straumann Standard Plus vs. Astra Tech EV), and measurement position (mesial, distal, buccal, lingual) as fixed factors and a random intercept for implant. Because each student placed exactly one implant, operator and implant coincide in the student group. Only the two experienced surgeons placed multiple implants. The interaction between surgical experience and implant system was included to assess whether the effect of experience differed between systems.

Estimated marginal means were derived from the model, and pairwise comparisons were performed with Bonferroni correction for multiple comparisons. The significance level was set at α = 0.05. Exact p-values are reported throughout; values below 0.001 are reported as *p* < 0.001.

## Results

**Fig. 2:**
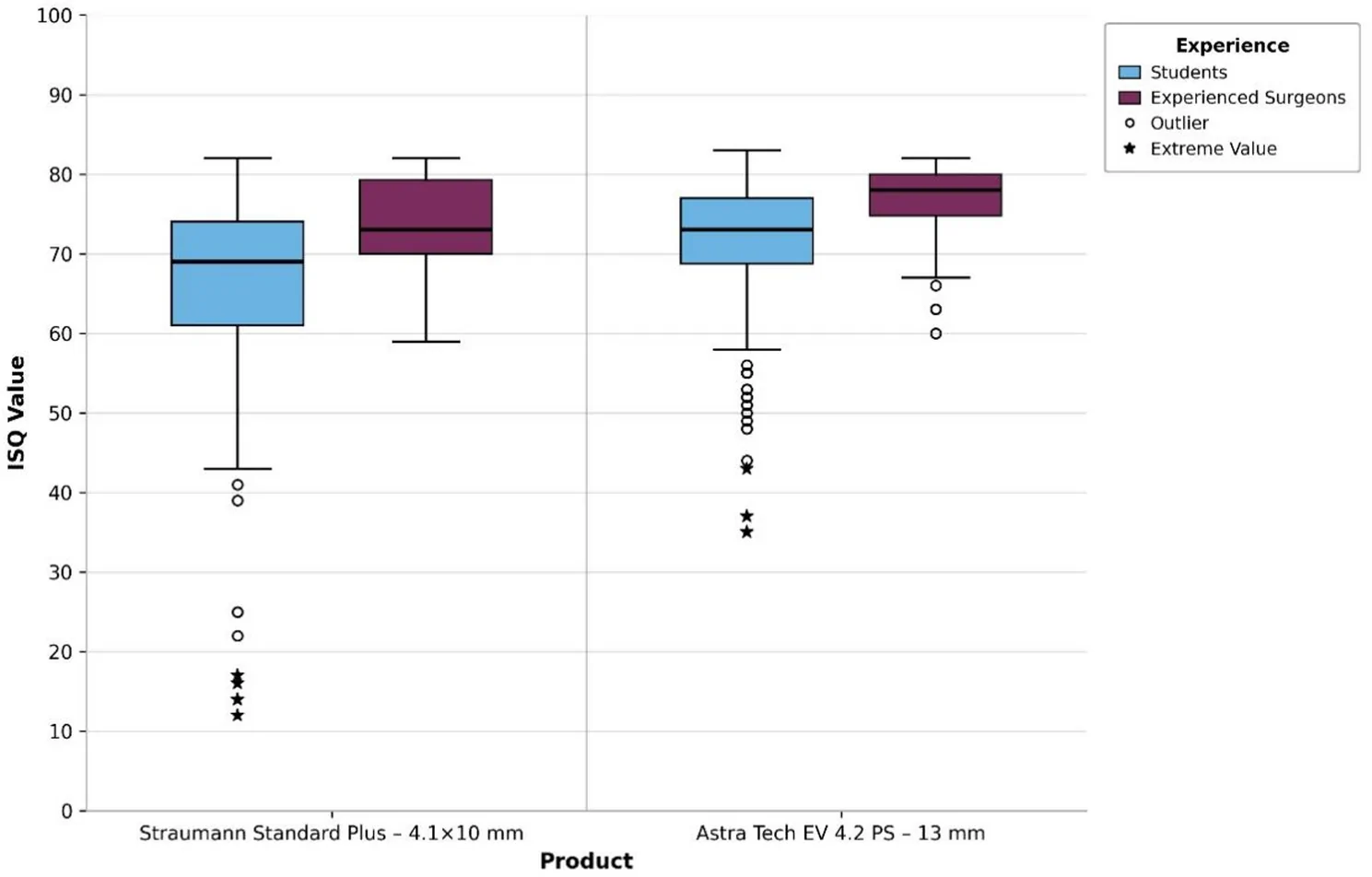
Key results. Boxplots of ISQ values stratified by surgical experience and implant system. Horizontal lines indicate medians; boxes represent interquartile ranges; whiskers extend to 1.5 × IQR; circles (○) denote outliers (> 1.5 × IQR); asterisks (★) denote extreme values (> 3 × IQR). Experienced surgeons achieved higher and more consistent ISQ values than students within both implant systems

A total of 760 ISQ measurements were included in the analysis (Straumann Standard Plus: *n* = 280; Astra Tech EV: *n* = 480). Descriptive statistics stratified by surgical experience and implant system are presented in Table 1. The mixed-effects model was statistically significant; the fixed effects accounted for 15.6% of the variance in ISQ (marginal R² = 0.156), rising to 88.3% when the random effects were included (conditional R² = 0.883), reflecting the strong clustering of the four measurements within each implant (intraclass correlation = 0.86). Key results are illustrated in Fig. 2.

**Table 1:** Descriptive Statistics of ISQ Values by Surgical Experience and Implant System

Group	*n*	Mean ISQ	SD	Range
**Straumann Standard Plus (4.1 × 10 mm)**				
Students	224	65.30	14.06	12–82
Experienced Surgeons	56	73.45	6.11	59–82
**Astra Tech EV (4.2 PS × 13 mm)**				
Students	316	71.08	8.76	35–83
Experienced Surgeons	164	76.49	4.65	60–82
**Overall**				
Students	540	68.68	11.61	12–83
Experienced Surgeons	220	75.72	5.22	59–82
**Total**	**760**	**70.72**	**10.67**	**12–83**

### Effect of surgical experience

Surgical experience had a significant and clinically most relevant effect on ISQ values in the model (χ²(1) = 16.91; *p* < 0.001). Experienced surgeons achieved consistently higher and more predictable ISQ values than students across both implant systems (estimated marginal mean difference: 6.22 ISQ points; 95% CI [3.25; 9.18]). The experience × implant system interaction was not significant (χ²(1) = 0.67; *p* = 0.412), indicating that the magnitude of the experience effect did not differ significantly between the two systems.

#### Straumann standard plus

Surgeons achieved a mean ISQ of 73.45 (SD = 6.11) compared to 65.30 (SD = 14.06) in students, a difference of 8.14 ISQ points (estimated marginal mean contrast; 95% CI [0.90; 15.38]; *p* = 0.028). The markedly wider spread in the student group (SD = 14.06 vs. 6.11) indicates substantially less consistent outcomes among inexperienced operators.

#### Astra tech EV

Surgeons achieved a mean ISQ of 76.49 (SD = 4.65) compared to 71.08 (SD = 8.76) in students, a difference of 5.42 ISQ points (estimated marginal mean contrast; 95% CI [2.76; 8.08]; *p* < 0.001). As with the Straumann system, variability was substantially greater in the student group.

### Effect of measurement position

Measurement position showed a small but significant effect (χ²(3) = 84.90; *p* < 0.001). Mesial and distal positions yielded slightly higher ISQ values than buccal and lingual positions (all pairwise *p* < 0.001, Bonferroni-corrected), while no difference was found within the axial pair (mesial vs. distal: *p* > 0.999) or the lateral pair (buccal vs. lingual: *p* > 0.999). This position effect was significant within both systems (Astra Tech EV: χ²(3) = 91.29, *p* < 0.001; Straumann Standard Plus: χ²(3) = 14.82, *p* = 0.002).

## Discussion

The present study demonstrates that surgical experience has a significant and clinically relevant influence on the primary stability of dental implants. Across all analyses, experienced surgeons consistently achieved higher ISQ values than students, with mean differences ranging from approximately 5 to 8 ISQ points. This effect remained consistent after adjustment for implant system and measurement position, although the associated effect size was small to moderate (standardized mean difference d ≈ 0.6), and the fixed effects of the model explained only 15.6% of the total variance in ISQ values. A substantial proportion of variability therefore remains attributable to other anatomical, biomechanical, or procedural factors not captured in this model.

These findings are well aligned with the theoretical framework outlined in the introduction. Primary stability is a central prerequisite for successful osseointegration and is substantially influenced by surgical, anatomical, and implant-specific factors [8, 10, 12, 13]. While material properties, implant design, and surface modifications have been extensively investigated and optimized over the past decades [8, 9], the present data specifically address the often less quantified contribution of surgical execution. The results support the assumption that operative factors — including implant bed preparation, assessment of bone quality, torque control, and three-dimensional positioning — directly influence initial mechanical stability [14–16].

A central aspect is the embedding of these results within the context of immediate and early loading concepts. These protocols require sufficient primary stability to prevent micromovements exceeding critical thresholds that may lead to fibrous rather than osseous integration [17–19]. Against this background, differences of several ISQ points are not merely statistical effects but potentially clinically relevant. Lower primary stability among less-experienced operators may result in implants falling below the threshold required for safe immediate or early loading more frequently. Commonly used clinical reference values consider ISQ values ≥ 70 favorable for immediate or early loading and values < 60 critical [17, 20, 21]. In the present data, the mean ISQ of the student group (68.68) lies close to this decision region, whereas that of the experienced surgeons (75.72) lies clearly above it. A mean difference of 5 to 8 ISQ points can therefore shift an implant across a clinically relevant threshold. In clinical practice, such differences might inform treatment decisions, for example, the choice of a delayed rather than an immediate loading protocol, although this remains a hypothesis that requires confirmation in clinical studies. It must be emphasized that no consensus minimal clinically important difference for the ISQ has been established, so the clinical weight of a given ISQ difference should be interpreted with corresponding caution. However, as this study assessed primary stability in an ex vivo model exclusively, no direct conclusions can be drawn regarding clinical endpoints such as osseointegration, implant survival, or actual loading failures. These observations should therefore be regarded as hypothesis-generating rather than as definitive clinical evidence. These findings are consistent with the ex vivo results of Romanos et al. [22], who demonstrated that experienced clinicians achieved significantly higher ISQ values than less experienced operators, specifically when placing tapered implants in poor-quality bone, and with the RFA data reported by Toyoshima et al. [23], who showed that drilling protocol significantly influences primary stability of the Straumann Standard Plus in porcine bone.

The descriptive analysis further revealed that ISQ values showed considerably greater variability among students than among surgeons. This indicates that outcomes achieved by less experienced operators are not only lower on average but also less consistent. In the context of immediate loading concepts, which rely on predictable primary stability, this increased variability may be of practical relevance.

The two implant systems included in this study differ in diameter, length, and macrogeometric design, precluding a direct comparison of ISQ values between the systems. In particular, the greater length (13 vs. 10 mm) and slightly larger diameter (4.2 vs. 4.1 mm) of the Astra Tech EV increase the bone–implant contact area and may independently raise ISQ values, irrespective of operator experience or macrogeometric design. For this reason, no comparison of absolute ISQ values was drawn between the two systems, and the experience effect was interpreted strictly within each system. Nevertheless, the observation that surgical experience had a significant and consistent effect within both systems underscores that operative execution is a decisive determinant of primary stability within both implant systems investigated. This suggests that even with modern, optimized implant systems, the quality of surgical technique remains a critical limiting factor — a finding consistent with previous clinical observations [10, 22, 24].

Within each system, simple effects analyses confirmed significantly higher ISQ values for surgeons than for students, supporting a general effect of surgical expertise rather than a system-specific phenomenon.

The significant effect of measurement position, though small in magnitude, aligns with anatomical considerations. Mesial and distal measurements showed slightly higher ISQ values than buccal and lingual readings, which may reflect differences in cortical bone thickness or instrument coupling across measurement axes. Given the small effect size, this factor appears to be of limited clinical relevance.

The use of porcine mandibles as a bone surrogate is an established model in implant research and offers the advantage of standardized conditions with bone density comparable to that of the human posterior mandible [8]. However, direct extrapolation to the clinical situation should be approached with caution, as factors such as patient-specific anatomy, variability in bone quality, soft-tissue management, and intraoperative conditions are not replicated in this ex vivo setting. In particular, the ex vivo model cannot reproduce intraoperative bleeding, patient movement, dynamic soft-tissue handling, or the biological processes of bone healing and osseointegration that ultimately determine implant survival. The present results are therefore restricted to the immediate mechanical situation at the time of placement and permit no conclusions regarding clinical endpoints. Furthermore, the study was conducted at a single center by a limited number of operators. This explains the pronounced imbalance between the two groups: 135 students versus only two experienced surgeons. As a consequence, the low variability observed in the surgeon group (SD = 5.22) reflects the repeated-measures behavior of these two specific individuals rather than the variability of experienced surgeons as a population, and the factor “surgical experience” is inevitably confounded with the personal technique, skill, and system familiarity of these two operators. This confound limits the findings’ generalizability. Strictly interpreted, the study demonstrates a difference between novices and two experienced benchmark operators rather than between novices and experienced surgeons as a group. In addition, a small number of very low ISQ values were recorded. These most probably reflect isolated placement failures, local bone fracture, or inadequate SmartPeg coupling rather than the typical performance of inexperienced operators, and they contribute disproportionately to the greater variance seen in the student group. Future studies should include a broader range of operators across multiple centers and should assess the influence of structured training programs on primary stability outcomes over time.

Taken together, the results support the hypothesis that surgical experience is a key determinant of primary implant stability. The data demonstrate that surgical expertise is one of the strongest identified factors, with a clinically relevant effect consistent across both implant systems. These findings are consistent with a potential benefit of structured, supervised training for novice operators, a hypothesis that warrants prospective clinical evaluation, and highlight that surgical technique remained a decisive factor within both implant systems investigated.

## Conclusions

The present study showed that surgical experience significantly influences primary implant stability, as measured by the ISQ via resonance frequency analysis. Experienced surgeons achieved consistently higher and more predictable ISQ values than dental students placing their first implants within both implant systems investigated. The experience effect was consistent across systems and represents a clinically relevant determinant of primary stability. Within the limits of this ex vivo model and its two-surgeon benchmark, these findings suggest that supervised training may help less-experienced operators achieve higher and more predictable primary stability, and that adapted loading protocols might be considered for novices, hypotheses that should be confirmed in clinical studies.

## Data Availability

The datasets generated and/or analysed during the current study are available from the corresponding author on reasonable request.

## Abbreviations

CI: Confidence Interval
ITV: Insertion Torque Value
ISQ: Implant Stability Quotient
RFA: Resonance Frequency Analysis
SD: Standard Deviation
SPSS: Statistical Package for the Social Sciences
